# Influence of Transcranial Direct Current Stimulation to the Cerebellum on Standing Posture Control

**DOI:** 10.3389/fnhum.2016.00325

**Published:** 2016-07-07

**Authors:** Yasuto Inukai, Kei Saito, Ryoki Sasaki, Shinichi Kotan, Masaki Nakagawa, Hideaki Onishi

**Affiliations:** ^1^Department of Physical Therapy, Niigata University of Health and WelfareNiigata, Japan; ^2^Institute for Human Movement and Medical Sciences, Niigata University of Health and WelfareNiigata, Japan

**Keywords:** transcranial direct current stimulation, cerebellum, standing posture control, center of gravity sway, vestibulospinal tract

## Abstract

Damage to the vestibular cerebellum results in dysfunctional standing posture control. Patients with cerebellum dysfunction have a larger sway in the center of gravity while standing compared with healthy subjects. Transcranial direct current stimulation (tDCS) is a noninvasive technique for selectively exciting or inhibiting specific neural structures with potential applications in functional assessment and treatment of neural disorders. However, the specific stimulation parameters for influencing postural control have not been assessed. In this study, we investigated the influence of tDCS when applied over the cerebellum on standing posture control. Sixteen healthy subjects received tDCS (20 min, 2 mA) over the scalp 2 cm below the inion. In Experiment 1, all 16 subjects received tDCS under three stimulus conditions, Sham, Cathodal, and Anodal, in a random order with the second electrode placed on the forehead. In Experiment 2, five subjects received cathodal stimulation only with the second electrode placed over the right buccinator muscle. Center of gravity sway was measured twice for 60 s before and after tDCS in a standing posture with eyes open and legs closed, and average total locus length, locus length per second, rectangular area, and enveloped area were calculated. In Experiment 1, total locus length and locus length per second decreased significantly after cathodal stimulation but not after anodal or sham stimulation, while no tDCS condition influenced rectangular or enveloped areas. In Experiment 2, cathodal tDCS again significantly reduced total locus length and locus length per second but not rectangular and enveloped areas. The effects of tDCS on postural control are polarity-dependent, likely reflecting the selective excitation or inhibition of cerebellar Purkinje cells. Cathodal tDCS to the cerebellum of healthy subjects can alter body sway (velocity).

## Introduction

Human balance is controlled by vestibular, visual, and somatosensory inputs to the brainstem and vestibular cerebellum (Peterka and Loughlin, 2004). The cerebellum is involved in motor learning and motor control, and patients with cerebellum dysfunction have a larger sway in the center of gravity while standing (Mauritz et al., 1979; Ilg et al., 2009). Both PET and fMRI show increased cerebellum activity while standing (Ouchi et al., 1999; Jahn et al., 2008), consistent with an important function in standing posture control. The cerebellar vermis is unique in that it projects bilaterally to the brain reticular formation and lateral vestibular nuclei via the deep cerebellar fastigial nucleus (Hans et al., 2014). In turn, reticular formation and lateral vestibular nuclei send projections via the reticulospinal tract and lateral vestibulospinal tract, respectively, to bilateral spinal motor nuclei that contribute to standing posture control.

Transcranial direct current stimulation (tDCS) is a noninvasive brain stimulation method known to increase or decrease excitation in the cerebral cortex depending on specific stimulus conditions (Priori, 2003). Nitsche and Paulus (2001) reported that anodal electrode stimulation to the primary motor area increased motor evoked potential (MEP) amplitude, an indicator of enhanced excitation in the corticospinal tract, while placing the cathodal electrode on the primary motor area decreased MEP amplitude. Thus, tDCS responses are clearly influenced by polarity (Nitsche and Paulus, 2001; Boros et al., 2008).

The effects of tDCS on other cortical regions have also been investigated. Parazzini et al. (2014) reported that 2-mA stimulation 2 cm below the inion results in current spread throughout the cerebellum with little spread to surrounding regions such as the brain stem according to computational analysis; tDCS to the cerebellum alters MEP amplitude and cerebello-brain inhibition (CBI; Galea et al., 2009), motor learning (Galea et al., 2011), and gait (Jayaram et al., 2012), but no previous study has assessed the effects of standing posture control. Therefore, exactly how modulation of cerebellum activity influences standing posture control and the optimal tDCS parameters for this purpose are unclear.

Galvanic vestibular stimulation (GVS) is a possible alternative intervention for postural control. When stimulating one mastoid by the anode and the other by the cathode, it is known that postural orientation or center of gravity will deviate to the anodal stimulation side (Day et al., 1997; Fitzpatrick and Day, 2004; Son et al., 2008). However, MacDougall et al. (2006) reported that the influence only lasts during stimulation, suggesting that although GVS is useful for assessing vestibular dysfunction, it may not be suitable for improvement of postural control or to prevent falls.

This study examined the influence of tDCS applied to the cerebellum on standing posture control as an initial step in assessing its potential to prevent falls and other risks associated with vestibular cerebellum dysfunction. In this study, we first calculated the total locus length as the center-of-gravity travel distance, the locus length per second as an index of center-of-gravity travel velocity, and the rectangular and enveloped areas as indices of center-of-gravity travel area before and after tDCS. We then tested whether tDCS to the cerebellum can affect standing posture control.

## Materials and Methods

### Subjects

Sixteen healthy male subjects (21.0 ± 2.9 years, mean ± standard deviation [SD]) participated in this study. None had a history of neuromuscular or cardiovascular diseases, and all gave their written informed consent to participate. The study conformed to the Declaration of Helsinki guidelines and was approved by the ethics committee of Niigata University of Health and Welfare, Japan.

### tDCS

Transcranial DCS was delivered using a DC-STUMULATOR (Eldith, NeuroConn GmbH, Germany) through a pair of saline-soaked surface sponge electrodes (5 × 7 cm, 35 cm^2^; current density: 0.057 mA/cm^2^). Direct current at 2.0 mA was applied for 20 min with fade-in/fade-out times of 5 s.

### Measurement of Center of Gravity Sway

Center of gravity sway was measured for 60 s at 20 Hz in the standing position with eyes open and legs closed using a Gravicorder G-5500 (Anima, Japan). Subjects stood for 10 s before the first of two measurement sessions separated by a 60-s rest period. Average total locus length, locus length per second, rectangular area, and enveloped area were calculated before and after tDCS.

### Experimental Procedures

This study consisted of two experiments. Experiment 1 was performed in all 16 subjects while Experiment 2 was conducted in a subgroup of 5 [age, 24.60 ± 2.61 (range: 23−29) years]. In Experiment 1, the center of the stimulation electrode was placed 2 cm below the inion and the other electrode on the forehead. In Experiment 2, the center of stimulation electrode was also placed 2 cm below the inion and the other electrode on the right buccinator muscle. In Experiment 1, three stimulus conditions were tested in random order: sham, cathodal, and anodal. In Experiment 2, only cathodal stimulation was tested based on the results of Experiment 1.

Following the two pre-tDCS measurements of the center of gravity sway, tDCS was conducted with the subject sitting quietly in a chair. The subject was blinded to stimulation condition and each particular condition in Experiment 1 was carried out at more than 3-day intervals. Experiment 2 was conducted more than 1 month after Experiment 1.

### Analysis

Statistical analysis was performed using PASW version 18 (SPSS; IBM, Armonk, NY, USA). In Experiment 1, mean total locus length, locus length per second, rectangular area, and enveloped area pre-tDCS and post-tDCS were compared by two-way repeated measures analysis of variance (ANOVA) [stimulus conditions (sham, cathodal, or anodal)] × [time (pre-tDCS or post-tDCS)]. For factors confirmed significant by paired Student’s *t*-test, if a parameter (total locus length, locus length per second, rectangular area, and enveloped area) differed significantly between pre-tDCS and post-tDCS measures, Pearson’s product-moment correlation coefficients were calculated for the pre-tDCS vs. pre-tDCS/post-tDCS ratio to clarify effects on tDCS response magnitude. Intraclass correlation coefficients (ICC_(1,2)_) between the two successive pre-tDCS and post-tDCS measurements were calculated to test the reliability (replicability) of the results. We used paired Student’s *t*-test to compare mean total locus length, locus length per second, rectangular area, and enveloped area between pre-tDCS and post-tDCS measurements in Experiment 2. Differences were considered significant at *p* < 0.05.

## Results

**Figure 1:**
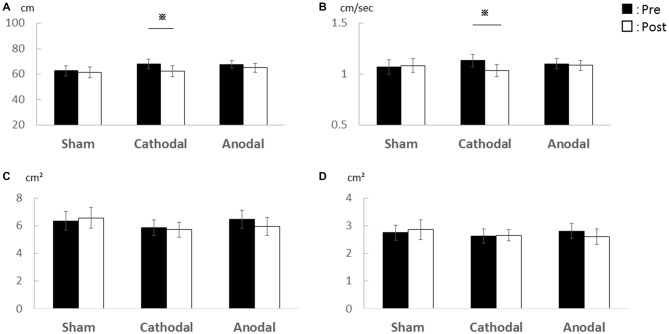
**Effects of transcranial direct current stimulation (tDCS) on the cerebellum on total locus length, locus length per second, rectangular area, and enveloped area. (A)** Total locus length. **(B)** Locus length per second. **(C)** Rectangular area. **(D)** Enveloped area. The total locus length and locus length per second were significantly lower after cathodal stimulation (*p* < 0.01) while rectangular area and enveloped area were unchanged. Error bars indicate SE.

In Experiment 1, there was a significant decrease in the total locus length and locus length per second after cathodal tDCS to the cerebellum (Figure 1). For total locus length, two-way repeated measures ANOVA revealed significant main effect of time (post-tDCS vs. pre-tDCS) [*F*_(1,15)_ = 6.68, *p* = 0.021] and a stimulus condition (Sham vs. Cathodal vs. Anodal) × time interaction [*F*_(1.22,18.37)_ = 4.17, *p* = 0.049], whereas the main effect of stimulation condition was non-significant [*F*_(1.81,27.32)_ = 0.04, *p* = 0.94]. Locus length per second exhibited non-significant main effects of both stimulation condition [*F*_(1.61,24.19)_ = 0.07, *p* = 0.898] and time [*F*_(1,15)_ = 4.44, *p* = 0.052]. However, the stimulus condition × time interaction was significant [F_ (1.28, 19.19)_ = 4.603, *p* = 0.037]. Rectangular area non-significant main effect of both stimulation condition [*F*_(0.91,3.06)_ = 0.298, *p* = 0.714] and time [*F*_(1,15)_ = 0.040, *p* = 0.844], the stimulus condition × time interaction, was non-significant [*F*_(1.13,1.37)_ = 0.825, *p* = 0.442]. Enveloped area non-significant main effect of both stimulation condition [*F*_(1.70,25.52)_ = 0.143, *p* = 0.835] and time [*F*_(1,15)_ = 0.219, *p* = 0.646], the stimulus condition × time interaction, was non-significant [*F*_(1.59,23.854)_ = 0.814, *p* = 0.430].

*Post hoc* pairwise comparisons with paired Student’s *t*-test revealed that post-tDCS total locus length differed significantly from the pre-tDCS value following cathodal stimulation (*p* = 0.001, *r* = 0.72) but not following sham (*p* = 0.846, *r* = 0.05) or anodal stimulation (*p* = 0.123, *r* = 0.39). Similarly, post-tDCS locus length per second differed significantly from the pre-tDCS value following cathodal tDCS (*p* = 0.003, *r* = 0.68) but not following sham (*p* = 0.835, *r* = 0.06) or anodal stimulation (*p* = 0.290, *r* = 0.27).

**Figure 2:**
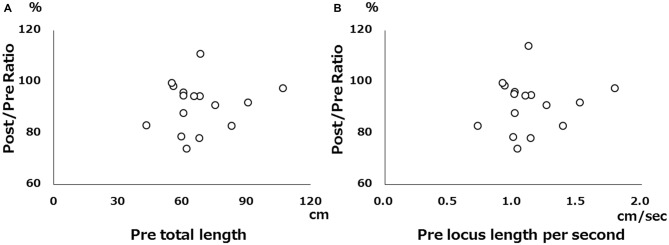
**Scatter diagram of pre-tDCS measurements vs. pre/post ratio. (A)** Scatter diagram of pre-tDCS total locus length vs. post/pre ratio. **(B)** Scatter diagram of pre-tDCS locus length per second vs. post/pre ratio. There were no significant correlations.

There was no significant correlation between pre-tDCS and post/pre ratio for either total locus length or locus length per second in the cathodal stimulation condition (Figure 2), indicating that baseline value did not influence the magnitude of the change following tDCS. However, both total locus length and locus length per second ICCs between the pre-tDCS first and pre-tDCS second measurements and the post-tDCS first and post-tDCS second measurements were around 0.9 under all conditions, consistent with high test-retest reliability (Table 1).

**Table 1:** **Intraclass correlation coefficients between the two replicate measurements conducted before (Pre) and after (Post) transcranial direct current stimulation (tDCS)**.

	Sham				Cathodal				Anodal			
	Pre		Post		Pre		Post		Pre		Post	
	ICC	95% CI	ICC	95% CI	ICC	95% CI	ICC	95% CI	ICC	95% CI	ICC	95% CI
Total locus length	0.90	0.720–0.965	0.91	0.737–0.967	0.88	0.665–0.958	0.90	0.720–0.965	0.87	0.635–0.954	0.89	0.702–0.962
Locus length per second	0.90	0.728–0.966	0.91	0.737–0.967	0.88	0.653–0.956	0.90	0.723–0.965	0.87	0.636–0.954	0.87	0.637–0.954
Rectangular area	0.74	0.272–0.908	0.86	0.618–0.952	0.55	−0.225–0.841	0.78	0.397–0.924	0.65	0.025–0.887	0.64	0.002–0.874
Enveloped area	0.76	0.372–0.921	0.89	0.681–0.960	0.76	0.342–0.917	0.72	0.224–0.902	0.65	0.014–0.875	0.79	0.426–0.927

*The ICC_(1,2)_ was calculated to test the reliability (reproducibility) of the measurement results. The ICCs for both total locus length and locus length per second were high (around 0.9) for all interventions. ICC, intraclass correlation coefficient (model 1, 2), 95% CI = 95% confidence interval*.

**Figure 3:**
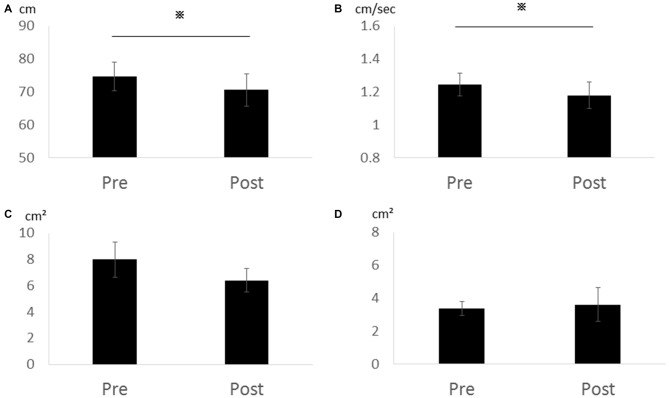
**Effects of cathodal tDCS to the cerebellum on total locus length, locus length per second, rectangular area, and enveloped area in Experiment 2. (A)** Total locus length. **(B)** Locus length per second. **(C)** Rectangular area. **(D)** Enveloped area. Total locus length and locus length per second were significantly reduced after cathodal tDCS (*p < 0.05). Error bars indicate SE.

In Experiment 2, total locus length (*p* = 0.028, *r* = 0.86) and locus length per second (*p* = 0.028, *r* = 0.86) decreased significantly after cathodal tDCS, while rectangular area *(p* = 0.295, *r* = 0.52) and enveloped area (*p* = 0.800, *r* = 0.13) were not changed (Figure 3). Thus, the effects appear to depend on the cathode position (over the cerebellum) rather than the position of the second electrode (forehead vs. right buccinator muscle).

## Discussion

We demonstrate that cathodal tDCS adjacent to the vestibular cerebellum reduce total locus length and locus length per second in the standing position, while anodal stimulation has no effect. Moreover, the high ICCs for total locus length and locus length per second (>0.9) indicate the strong replicability of these measures (Landis and Koch, 1977). This effect was observed whether the second electrode was on the forehead (Experiment 1) or the right buccinator muscle (Experiment 2), indicating that these decreases depend on the stimulating electrode position near the vestibular cerebellum.

A previous modeling study reported that tDCS at the inion stimulated a wide region of the cerebellum with tDCS of the brain stem (Parazzini et al., 2014), consistent with our results that cathodal tDCS to the inion can influence standing posture control. Nitsche et al. (2003) and Di Lazzaro et al. (2012) reported that cathodal tDCS to the primary motor area decreased excitation, implying that cathodal tDCS may decrease total locus length and locus length per second by suppressing Purkinje cell activity in the cerebellar cortex.

Ugawa et al. (1991, 1995) reported that the MEP amplitude in response to electric or magnetic stimulation of the primary motor cortex (M1) was reduced when preceded 5−7 ms earlier by magnetic stimulation of the cerebellum compared to M1 stimulation alone, an effect referred to as CBI. It is thought that proceeding stimulation to the cerebellum stimulates GABAergic Purkinje cells, which in turn transiently inhibit the output of the cerebellar dentate nucleus−thalamus−motor cortex pathway, resulting in a temporary decrease in M1 excitation (Pinto and Chen, 2001; Daskalakis et al., 2004). Alternatively, Galea et al. (2009) reported that cathodal stimulation of the right cerebellar cortex markedly reduced CBI as measured by (elevated) MEPs in response to transcranial magnetic stimulation (TMS) of M1, but had no effect on short latency intracortical inhibition (SICI) or short latency intracortical facilitation (ICF) in response to paired magnetic stimulation of M1.

Purkinje cells form the only descending fiber output from the cerebellar cortex. These axons are GABAergic, thus inhibiting the deep cerebellar nuclei. Cathodal tDCS thus likely inhibits Purkinje cells and decreases inhibition of deep cerebellar nuclei (Grimaldi et al., 2014), thereby enhancing excitatory outputs to M1. The same mechanism may also explain our results. If cathodal tDCS inhibits Purkinje cells, inhibition of the cerebellar fastigial nucleus would be reduced. This would disinhibit the brain stem reticular formation and lateral vestibular nucleus, which could then excite reticulospinal and lateral vestibulospinal tracts. It is thought that reticulospinal and vestibulospinal tracts control the level of posture muscle tone (Takakusaki, 2013). We speculate that cathodal tDCS alters the excitability of reticulospinal and vestibulospinal tracts and thus postural muscle tone, thereby changing the post-tDCS body sway.

Although there was a decrease in total locus length, no significant changes were observed in the rectangular area and enveloped area following cathodal tDCS. As there was also a decrease in locus length per second, the decrease in total locus length could reflect a decrease in the center of gravity velocity. In tDCS studies on lower limb M1, oscillation area does not significantly change, but velocity changes (Lazzari et al., 2015). The change in velocity may be a more sensitive parameter of neural changes induced by tDCS than change in the sway area.

Also, the fact that there was no relationship between pre-tDCS total locus length or pre-tDCS locus length per second and the associated pre/post ratio suggests that the magnitude of the changes are related more to individual factors than to the baseline values. Standing posture is predominantly controlled by three sensory modalities, vestibular, visual, and somatosensory, and the degree of dependence on each varies across individuals (Kluzik et al., 2005; Isableu and Vuillerme, 2006). Thus, the different contributions of each sense may affect the change ratio. In addition, it was reported that the difference in genotype and skull thickness alters the effect of tDCS on the M1 (Antal et al., 2010; Opitz et al., 2015; Puri et al., 2015). These factors may have influenced the stimulatory effect.

On the other hand, there were no significant effects of anodal tDCS on these center of gravity sway parameters. It was reported that anodal tDCS to cerebellum promoted motor learning and gait adaptation (Galea et al., 2011; Jayaram et al., 2012). In addition, it is reported that cerebellar activity increases during the standing posture in a standing posture (Ouchi et al., 1999; Jahn et al., 2008). In this study, however, all the subjects were healthy men, and the degree of difficulty of the task was low (standing with eyes open and legs closed). The absence of any need to learn or adapt may have contributed to the lack of effect of anodal tDCS. As a result, anodal tDCS did not affect standing posture control of healthy subjects.

One of the limitations of this study is that all subjects were healthy young men rather than patients with cerebellar dysfunction. The main finding of this study is that center of gravity unrest changed after cathodal tDCS in healthy young men. However, it is unclear whether the patients with cerebellar dysfunction and elderly people are similar to this results. Further study is required to apply the same experimental procedures to elderly patients or those with unstable standing posture control.

It is also unclear whether the decreases in total locus length and locus length per second after cathodal tDCS to the cerebellum have beneficial or deleterious effects on standing posture control. Although both total locus length and locus length per second decreased significantly after tDCS, it is unknown whether these changes imply an improvement in stability. Thus, it is necessary to assess postural parameters in addition to total locus length and locus length per second in future studies. In addition, it is not clear whether these changes arise from stimulation of the cerebellar vermis and cerebellar hemisphere or only from stimulation of the vermis. This issue can be addressed in future studies by changing the size and position of the electrodes. The present study was a single blind trial. Although we used quantitative parameters of postural control, a future double-blind trial may provide further validation.

The results suggest that tDCS improves tone of postural muscles and standing posture control by altering cerebellar excitability and activity of the reticulospinal and vestibulospinal tracts. tDCS to the cerebellum may contribute to the improvement of postural stability in cerebellar disorders and other cases of unstable standing posture, such as in the elderly.

In conclusion, we found out that cathodal tDCS to the cerebellum influences the total locus length and locus length per second during open-eyed standing. This indicates that tDCS to the cerebellum can influence standing posture control depending on polarity. Future tDCS studies are needed to elucidate the cerebellar mechanisms for these effects. It is possible that tDCS may have clinical efficacy for the improvement of standing posture control in the elderly and cerebellar disorder patients.

## Author Contributions

HO conceived the study and designed the experiment. YI conducted the experiments. KS, RS, and SK performed the interpretation of data. YI and MN performed the statistical analysis. SK, RS, and MN helped writing and revising the manuscript. HO and YI wrote the manuscript. All authors read and approved the final manuscript.

## Funding

This work was supported by a Grant-in-Aid for Exploratory Research from the Niigata University of Health and Welfare, 2015.

## Conflict of Interest Statement

The authors declare that the research was conducted in the absence of any commercial or financial relationships that could be construed as a potential conflict of interest.
